# Identification of Mechanical Parameters of the Silicon Structure of a Capacitive MEMS Accelerometer

**DOI:** 10.3390/ma18245676

**Published:** 2025-12-17

**Authors:** Kamil Kurpanik, Klaudiusz Gołombek, Edyta Krzystała, Jonasz Hartwich, Sławomir Kciuk

**Affiliations:** 1 Department of Theoretical and Applied Mechanics, Faculty of Mechanical Engineering Technology, Silesian University of Technology, Konarskiego 18A, 44-100 Gliwice, Poland; edyta.krzystala@polsl.pl (E.K.); jonasz.hartwich@polsl.pl (J.H.);; 2 Materials Research Laboratory, Faculty of Mechanical Engineering Technology, Silesian University of Technology, Konarskiego 18A, 44-100 Gliwice, Poland; klaudiusz.golombek@polsl.pl

**Keywords:** MEMS accelerometer, numerical modeling, dynamic analysis, MATLAB Simulink, model validation

## Abstract

The aim of this study was to conduct an advanced analysis of the MEMS sensor, including both experimental tests and numerical simulations, in order to determine its mechanical properties and operational dynamics in detail. It is challenging to find publications in the literature that are not based on theoretical assumptions or general manufacturer data, which do not reflect the actual microstructural characteristics of the sensor. This study uses a numerical model developed in MATLAB/Simulink, which allows the experimentally determined material characteristics to be combined with predictive dynamic modelling. The model takes into account key mechanical parameters such as stiffness, damping and response to dynamic loads, and the built-in optimisation algorithm allows the structural parameters of the MEMS accelerometer to be estimated directly from experimental data. In addition, SEM microscopic studies and EDS chemical composition analysis provided detailed information on the sensor’s microstructure, allowing its impact on mechanical properties and dynamic parameters to be assessed. The integration of advanced experimental methods with numerical modelling has resulted in a model whose response closely matches the measurement results, which is an important step towards further research on design optimisation and improving the reliability of MEMS sensors in diverse operating conditions.

## 1. Introduction

Over the last few decades, we have witnessed dynamic development in the field of sensor and actuator systems. Advances in manufacturing technology have enabled their production with significantly greater precision and quality [1], while also allowing for their miniaturisation, laying the foundations for entire industries such as the Internet of Things (IoT). A key achievement in this area was the creation of microelectromechanical systems (MEMS), which form the basis of today’s actuators and sensors [2,3,4]. MEMS are defined as electromechanical systems made from components measuring between 1 and 100 μm, with total dimensions not exceeding 1 mm [5]. Systems of this type are characterised by low energy consumption, and in some applications even allow for energy recovery. This enables virtually autonomous operation of sensor systems [6]. Thanks to these properties, the use of MEMS will enable the creation of small mobile devices that form the basis of data acquisition systems that are part of the IoT, known as wireless sensor nodes (WSNs). MEMS often form the basis of WNS, not only by recovering electrical energy [7,8,9,10], but also by providing a system for measuring specific quantities. In consumer devices, MEMS are responsible, among other things, for measuring acceleration, which allows, among other things, the detection of the orientation of the device and the triggering of the airbag during a car collision. In the domain of passenger cars, MEMSs find application in a variety of functions, including the sensing of tyre pressure, brake fluid pressure, and seat belt closure [11]. Accelerometers are commonly used MEMS devices in consumer, industrial and research applications [12,13,14]. MEMS accelerometers used in these applications have many advantages, such as small size, low power consumption and easy integration with various data acquisition systems [15]. Due to their portability and versatility, MEMS accelerometers are also used in the study of rapidly changing quantities, for example, in car crash tests [16] and in studies of the impact of shock waves on the human body [17]. MEMS accelerometers are essentially capacitive devices consisting of a suspended pendulum-like proof mass assembly. In capacitive accelerometers, the displacement of the proof mass instigates an alteration in the capacitance of the capacitor. This, consequently, enables the acceleration to which the measuring system and the component to which it is attached are subjected to be determined [18]. MEMS accelerometers, like other sensor systems, are manufactured using highly precise and complex production methods such as photolithography, chemical processing and laser processing [19]. The use of precise production methods enables the repeatable manufacture of highly precise micro-scale systems. Due to the complex processing and small size of MEMSs, modelling their operation is a process as complex as their production. Manufacturers of MEMS accelerometers do not provide information on parameters that are key to the model, such as the value of the test mass and the parameters of the springs to which this mass is attached. Therefore, in order to develop a precise model that reflects the mechanical system of a capacitive MEMS accelerometer, it is necessary to conduct appropriate research. The standard used in many scientific publications to assess the quality and test the parameters of systems on this scale is research using a scanning electron microscope (SEM) [20,21,22,23,24]. R. Gholamzadeh et al. described the manufacturing process of a MEMS accelerometer using sequential and pulsed-mode DRIE processes [25]. The authors observed the manufacturing process using SEM. A. Aydemir et al. used SEM to visualise the proof mass of a capacitive accelerometer manufactured using a proprietary production process [26]. Numerical studies of MEMSs are a complex process, which is why various approaches to modelling the operation of this type of accelerometer can be found in scientific literature [27,28,29,30,31,32]. In their paper, R. Mukhiya et al. presented the design and numerical testing process for a DRIE-based MEMS capacitive accelerometer [27]. The simulation was performed using dedicated MEMS+^®^ software and MATLAB Simulink. Using MEMS+ software, the authors performed, among other things, modal, harmonic and Pull-In simulations to determine the acceptable operating voltage values for the developed accelerometer. The MATLAB environment was used primarily in system-level simulations to determine the change in capacitance at the accelerometer output as a result of various simulated forces. The authors compared the results obtained with the analytical method and found a high degree of consistency. V. Benevicius et al. identified the parameters of a capacitive accelerometer based on numerical simulation using data on human body accelerations during physical activity [32]. The authors developed a model of this type of accelerometer using Comsol software, employing MATLAB software to perform the simulation. The study showed that the relationship between acceleration (which is the force in the developed model) and displacement (which is the result of the simulation) is linear, confirming that such a design is feasible in a MEMS accelerometer. The model was validated based on data from observations of the movement of a uniaxial vibration stand.

The aim of the study was to conduct an advanced analysis of the MEMS sensor, including both experimental tests and numerical simulations, in order to determine its mechanical properties and operational dynamics in detail. It is challenging to find publications in the literature that are not based on theoretical assumptions or general manufacturer data, which do not reflect the actual microstructural characteristics of the sensor. This study uses a numerical model developed in MATLAB/Simulink, which allows the experimentally determined material characteristics to be combined with predictive dynamic modelling. The key design parameter—proof mass—was determined on the basis of microscopic measurements. On this basis, a mathematical model of the accelerometer was developed in the MATLAB environment. Validation was performed by comparing the simulation results with experimental data, achieving high consistency. In addition, EDS (Energy Dispersive Spectroscopy) analysis was used to determine the material composition of the sensor components. The developed model enables the prediction of the dynamic response of the ADXL377 accelerometer under simulation conditions, streamlining the process of experiment planning and measurement result verification.

## 2. Materials

**Figure 1:**
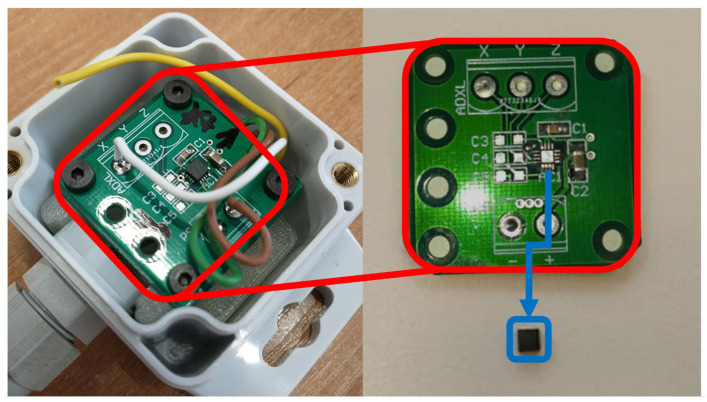
View of the MEMS sensor in its operational enclosure and the extracted PCB with the highlighted desoldered sensing component. The PCB board is presented in a red frame, while the disassembled MEMS sensor is presented in a blue frame.

The research conducted as part of the study focused on MEMS sensors, which are extremely precise devices used in a variety of fields, from industrial automation to medicine. These sensors are microscale structures integrating mechanical elements with electronics, enabling the measurement of parameters such as force, pressure and acceleration. Both the design and the materials used in their production are crucial to their functionality, performance and durability. The MEMS sensor tested was an object consisting of precision mechanical structures integrated with electronic circuits. Figure 1 shows the tested MEMS ADXL377 sensor (Analog Devices, Wilmington, MA USA) [33] in its housing.

**Figure 2:**
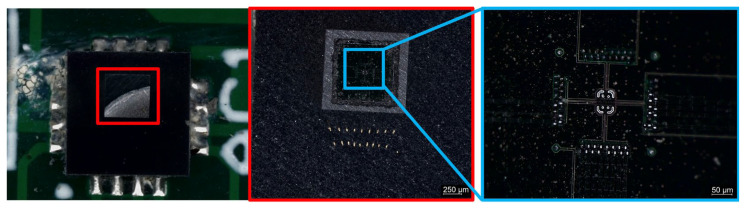
MEMS sensor opening process. The first magnification level is marked in red, and the second in blue.

Due to their small size and advanced manufacturing technology, MEMS sensors are subject to a number of challenges, such as microscale defects, contamination, and structural changes that can affect their properties. For this reason, it was very important to conduct a detailed material analysis to thoroughly understand how the structure and material composition affect the performance of these devices under various operating conditions. Figure 2 shows the successive stages of the mechanical removal of the MEMS sensor housing, carried out using abrasive discs used for material preparation. This procedure enabled the gradual exposure of the internal elements of the sensor structure, including silicon layers and conductive connections. The process of opening the sensor was necessary to perform further tests using scanning microscopy and elemental composition analysis, which provided detailed information about the structure and material of the tested system.

**Figure 3:**
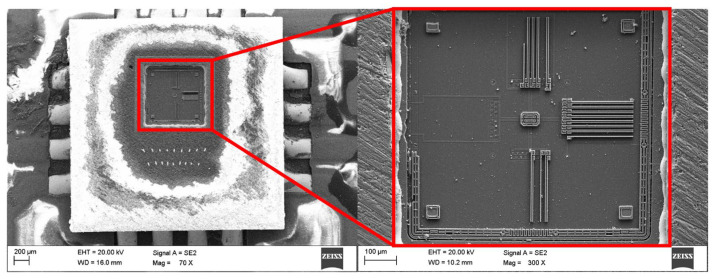
SEM images showing the surface of an open MEMS sensor.

High-resolution scanning electron microscopy (HRSEM) Supra 25 (ZEISS AG, Oberkochen, Germany) was used to analyse the surface of the MEMS sensor, enabling very high-resolution images to be obtained and detailed examination of its microstructure. The observations were made using a secondary electron (SE) detector at magnifications ranging from 70× to 300×. The use of HRSEM allowed for the characterisation of the material on a micro- and nanometric scale, which was necessary to identify potential imperfections that could affect the sensor’s performance. X-ray energy dispersive spectroscopy (EDS) complemented the SEM studies, allowing the local chemical composition of the MEMS sensor material to be determined. This technique facilitates the identification of elements through the analysis of the characteristic X-rays that are emitted by the sample as a consequence of electron bombardment. The use of SEM microscopy enabled the analysis of surface topography and the identification of structural defects such as cracks, discontinuities and damage to structural layers. The images obtained formed the basis for further comparative analyses with EDS results, conducted using an EDAX Trident spectrometer (XM4, AMETEK Inc., Berwyn, PA, USA). The integration of the results obtained from both methods allowed for a comprehensive structural and material characterisation of the MEMS sensor under investigation. Figure 3 shows an SEM image of the surface of an open MEMS sensor obtained during microstructural testing.

**Figure 4:**
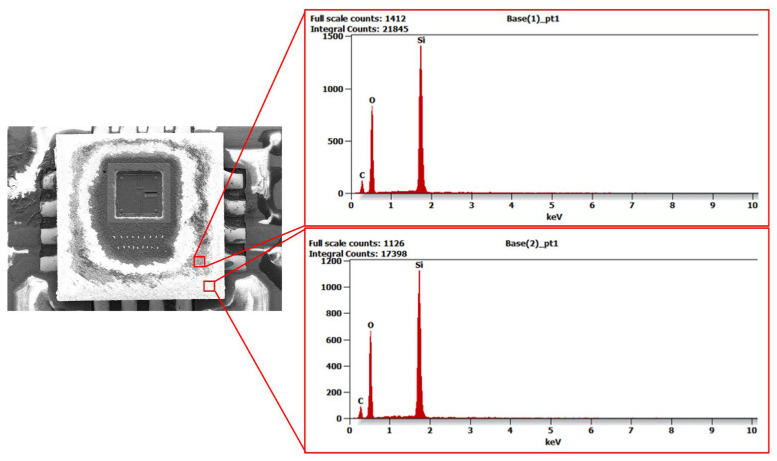
EDS analysis results showing the distribution of elements within the MEMS sensor housing.

**Figure 5:**
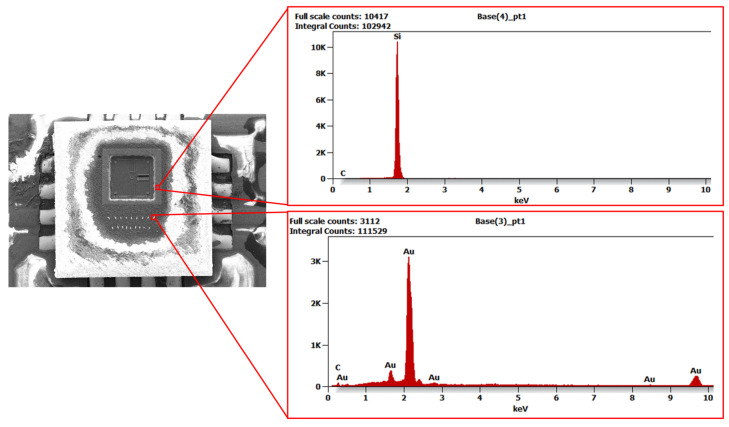
EDS analysis results showing the distribution of elements in the silicon structure envelope and in the MEMS sensor signal line.

**Figure 6:**
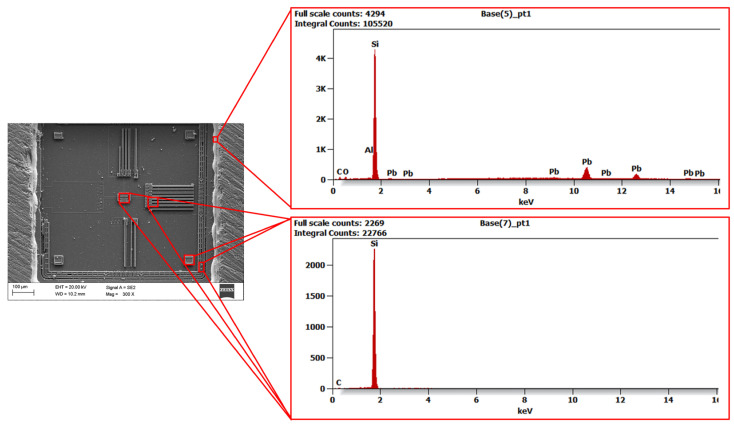
EDS analysis results showing the distribution of elements within the silicon structure of the MEMS sensor (magnification of the active area).

The image shows details of the surface topography and structural elements of the sensor. Thanks to the high resolution of this technique, it was possible to identify key surface features and any defects. Unfortunately, damage to part of the silicon structure is visible on the surface, which was caused by mechanical processing during the sensor opening process. This sensor was suitable for surface analysis. However, it should be emphasised that the opened sensor is not suitable for reuse in measurements, as its mechanical integrity was compromised during sample preparation. The images of the structure obtained in HRSEM provided important information about the surface condition and material characteristics, which is crucial for understanding the properties of MEMS sensors and the impact of potential defects on their performance. Figure 4, Figure 5 and Figure 6 show the X-ray diffraction spectra along with a qualitative analysis of the chemical composition and the area that was analysed.

The EDS analysis identified elements such as silicon and silicon oxide, which are the main components of the MEMS sensor structure. The presence of carbon in the spectrum is due to contamination during mechanical processing or sample preparation. In addition, gold was detected in the signal line area and a thin layer of lead surrounding the silicon structure, which may affect the electrical and mechanical properties of the sensor. The results obtained from SEM and EDS analyses were used as input data for mathematical modelling, allowing the microstructure and material composition of the sensor to be taken into account in the calculations. This enabled a better representation of its actual mechanical behaviour and an assessment of the impact of individual structural elements on the dynamic response of the system. The inclusion of material data in the model increased the accuracy of numerical analyses and enabled the assessment of potential areas of damage during operation.

## 3. Methods

As part of experimental research, the MEMS sensor was subjected to harmonic excitation generated by a laboratory shaker. The excitation was implemented in the form of a sinusoidal signal with a frequency ranging from 10 Hz to 50 Hz, with a step of 5 Hz. For each frequency value, the output signal from the MEMS sensor was recorded, along with data from reference sensors, which served as a reference point for comparative analysis. The recorded time histories were then used to develop dynamic characteristics and to calibrate and verify the numerical model of the sensor developed in the MATLAB environment.

The research station, which was designed and constructed as part of this project, was equipped with a set of laboratory devices that enabled precise dynamic measurements and control of the operating conditions of the MEMS sensor. The system included an input/output card responsible for transmitting control signals to the amplifier and then to the oscillator generating a controlled mechanical stimulus. The MEMS sensor was mounted on a shaker, which enabled the excitation of vibrations with specified frequency and amplitude parameters. In order to verify the correctness of the measurements, signals from the MEMS sensor were recorded in parallel with data from reference sensors (PCB Piezotronics 333B31 (PCB Piezotronics, Walden Ave, NY, USA) [34] and 352C33 (PCB Piezotronics, USA) [35]), which served as a reference point for analysing the accuracy and sensitivity of the tested system. The entire measurement system was designed to ensure stable experimental conditions, minimise interference and maximise signal acquisition resolution.

**Figure 7:**
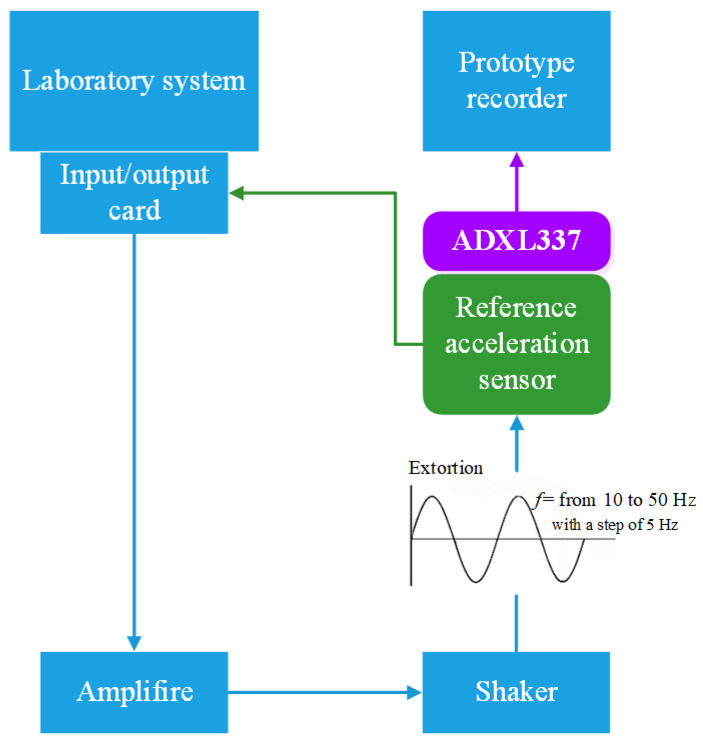
Diagram of the test rig used to test the MEMS sensor.

**Figure 8:**
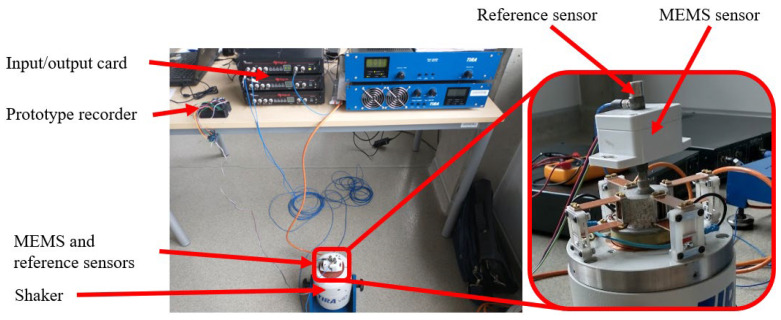
Actual workstation in the laboratory.

With the designed test rig, it was possible to obtain the actual response of the MEMS sensor to external forces, which was a key part of further numerical research. The obtained measurement data was used to calibrate and verify the mathematical model of the sensor, as well as to evaluate its performance in real conditions. Figure 7 shows a diagram of the test stand, illustrating the main components of the measurement system and how it works. Figure 8 shows the actual test stand, presenting the physical configuration of the devices used in the laboratory.

**Figure 9:**
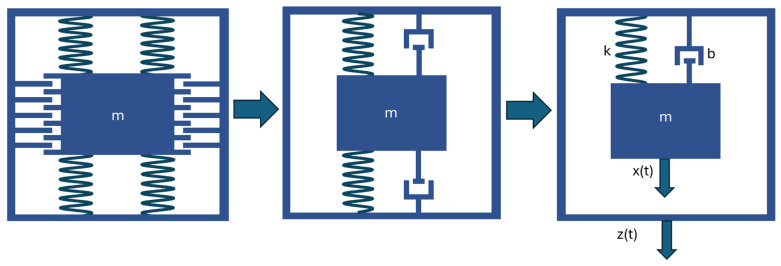
Modelling process diagram.

Mathematical modelling is a fundamental tool for analysing MEMS sensors, enabling the description of their behaviour in response to applied mechanical forces. The developed mathematical model of the dynamic system allows for the quantitative determination of the relationship between the external force and the sensor response, taking into account the elastic, damping and inertial properties of its components. The descriptive model defined in this way enables the analysis of the dynamic characteristics of the sensor under conditions close to real-life conditions and provides a basis for the calibration and verification of the results obtained during experimental tests. Figure 9 shows a simplified version of the MEMS sensor structure, in which the complex mechanics of the device are represented as a mass placed on a damper and spring.

This simplification allows for a dynamic analysis of the sensor, focusing on its behaviour in response to external forces. The equations of motion that describe the dynamics of this mechanical system are presented below. This model allows for further research into the sensor’s response to external variables, such as acceleration, and enables the prediction of its response under operating conditions. The equation of motion represents the dynamics of a simplified MEMS sensor model, where it is assumed that the system consists of a mass m, a spring with a spring constant k, and a damper with a damping coefficient b. Equations (1) and (2) of motion of the system is written as:

(1) mẍ=k(z−x)+b(ż−ẋ)

(2) $$ẍ=\frac{k\left(z-x\right)}{m}+\frac{b\left(ż-ẋ\right)}{m}$$

where

*m*—body mass [kg]

ẍ—body acceleration [m/s^2^]

*k*—spring stiffness [N/m]

*b*—damping coefficient [kg/s]

*x*—body displacement [m]

ẋ—body velocity [m/s]

*z*—reference point displacement [m]

ż—reference point velocity [m/s]

Equation (1) represents the general form of the equation of motion of the sensor mass in response to an external force, taking into account the effects of elasticity and damping. In order to obtain a more useful form for kinematic analysis of the system, this equation was transformed by dividing both sides by mass (*m*), which leads to Equation (2). This expression describes the acceleration of the sensor mass as a function of external excitation and the dynamic parameters of the system, allowing its behaviour under operating conditions to be assessed.

Numerical simulations are a key tool in analysing the dynamics of MEMS sensors, enabling the prediction of their behaviour under various operating conditions without the need for costly physical testing. The aim of the research was to determine the mechanical properties of MEMS sensors, such as stiffness, damping and response to mechanical forces. Numerical simulations were performed in the MATLAB Simulink environment, which is one of the most powerful tools for modelling dynamic systems. The MEMS sensor model in this environment allowed for the analysis of its response to mechanical forces and interactions between individual sensor components. The model took into account key mechanical parameters such as stiffness and damping. Stiffness was analysed in the context of the elastic properties of the sensor materials, and damping was analysed as the ability to reduce vibrations that could affect the accuracy of measurements. Mechanical excitation was a key variable in the simulation process, allowing the simulation of the sensor’s response to physical external stimuli. Thanks to the use of an optimisation algorithm, the numerical model enabled the analysis of the sensor’s behaviour in various load scenarios, which allowed its response to be precisely adjusted to actual operating conditions. As part of the optimisation process, key model parameters were determined, including stiffness and damping coefficients, whose values ensure that the simulation is consistent with the results of experimental measurements. The parameters were optimised using the particle swarm algorithm.

**Figure 10:**
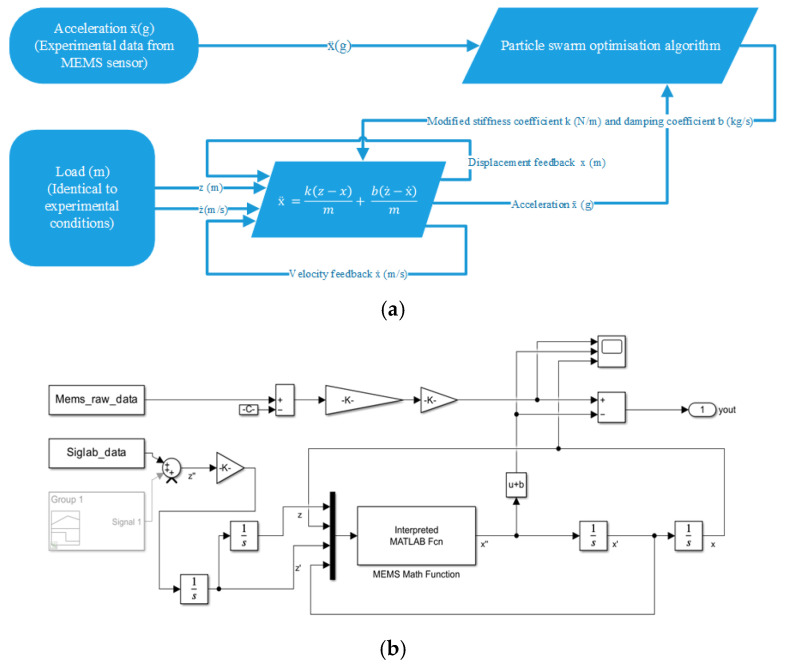
Presentation of: (**a**) schematic diagram of the numerical model of the MEMS sensor, (**b**) graphical representation of the model in the MATLAB Simulink environment.

An important element of the analysis was combining the SEM and EDS results with a numerical model. SEM and EDS tests allowed for the identification of the material composition of the MEMS sensor. The analysis of EDS spectra (Figure 4, Figure 5 and Figure 6) provided information about the microstructure of the material, including the presence of elements such as silicon and its oxides, which have a key impact on its mechanical properties. Figure 10a presents a block diagram illustrating the functional structure and data flow of the numerical model of the MEMS accelerometer. It outlines the key components of the system and their interactions, providing an overview of the model’s operating logic. Figure 10b shows the implementation of this model in the MATLAB Simulink environment, representing the dynamic behaviour of the sensor, including the motion of the proof mass as well as the effects of stiffness and damping. The developed model enables detailed analysis of the sensor’s response to external mechanical excitation and supports the optimisation of its performance under real operating conditions.

This model consists of several key blocks that enable the analysis of the sensor’s response to mechanical forces. The input blocks are responsible for entering data from an experiment conducted on a real MEMS sensor, which allows the results obtained to be compared with reference sensors. The input signal for the numerical model corresponded to the acceleration measured by the reference sensors acting on the MEMS sensor package, while the MEMS sensor response used for model validation was derived from the actual readings of the MEMS accelerometer. The block containing the formula describing the mechanics of seismic mass motion is used to map the dynamics of the sensor system, taking into account stiffness, damping and response to external stimuli. The integration and differentiation blocks enable the calculation of the system’s response over time, simulating the dynamic responses of the sensor to changing forces. The model integrates data from the experiment, making it possible to compare theoretical results with actual measurements, which allows for verification of the numerical model’s accuracy.

## 4. Results and Discussion

The results of numerical simulations are an important stage in the analysis of MEMS sensor dynamics. The computational model used enabled quantitative mapping of its response to mechanical forces and assessment of the impact of design parameters on the dynamic characteristics of the system. Preliminary simulations, performed at a sampling frequency of 300 Hz, showed that the acceleration waveforms were consistent with the reference data, with a deviation of 10%, which allowed for preliminary calibration of the model. After optimising the measurement system, including increasing the sampling frequency to 65 kHz and the measurement resolution to 0.8 mV/quantum, more accurate results were obtained, enabling a more precise representation of the sensor characteristics. This frequency refers to the speed of data recording from the analogue-to-digital converter and is not related to the excitation signal. The model used enabled the analysis of the influence of damping, stiffness and type of excitation on the dynamic response of the sensor. The results obtained confirmed the correctness of the model assumptions and increased its usefulness in further design analyses and in predicting the behaviour of MEMS sensors under variable operating conditions.

**Figure 11:**
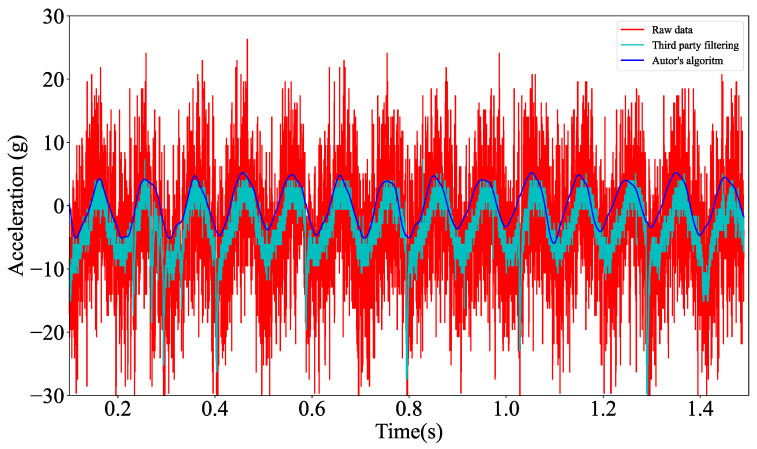
Sample waveform of measurement data comparing signal filtration results.

The graph (Figure 11) shows three sets of data illustrating different stages of signal processing:

- Red graph—represents raw measurement data from the MEMS sensor, without any filtering. This signal may contain interference, noise and other undesirable effects that affect its quality.
- Cyan graph—shows data after filtering using an external (third-party) system, which implements a moving average algorithm combined with a hardware low-pass filter. This filtering reduces some interference, but its operation may alter the signal characteristics, potentially affecting its correspondence with the actual behaviour of the sensor.
- Blue graph—data after filtering using our own system. This filtering, which combines a hardware low-pass filter, a digitally windowed using a Tukey window, and a digital Butterworth filter implemented via the Python SciPy module, provides a better representation of the actual behaviour of the sensor and is more consistent with the reference results, demonstrating the effectiveness of the proposed filtering approach.

**Figure 12:**
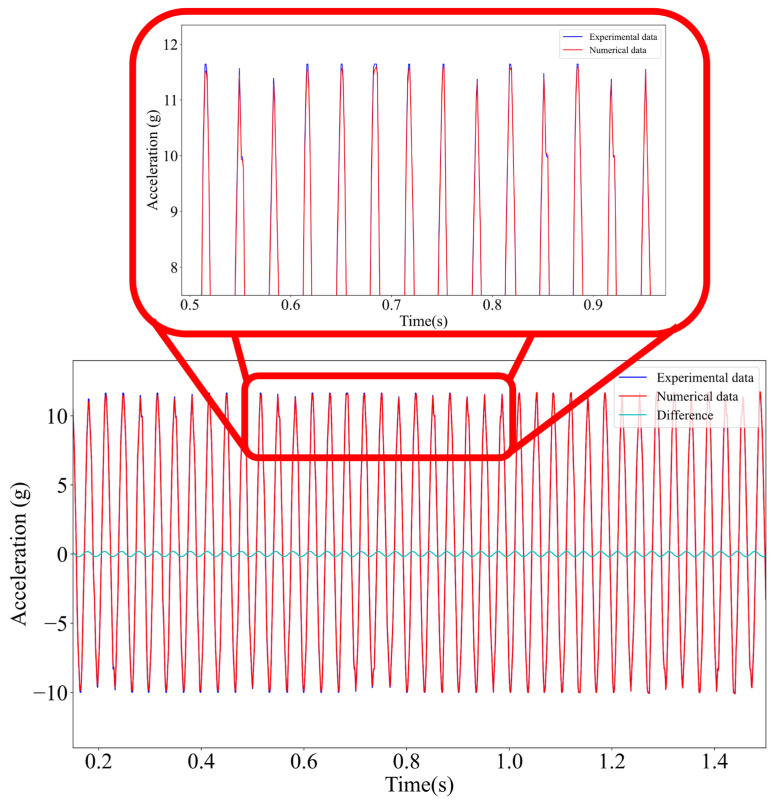
Waveform comparing the results of numerical simulations and actual measurements.

Figure 12 shows an example of a slight difference between the results of the numerical model and actual measurements. The differences between the theoretical and experimental data are small but noticeable, likely arising from numerical rounding, measurement noise, and minor experimental uncertainties. Despite these deviations, the overall agreement remains strong, confirming both the reliability of the numerical model and the accuracy of the experimental measurements.

As previously mentioned, the key design parameter—the proof mass—was determined on the basis of microscopic measurements and is equal to:

*m* = 5.528 × 10^−12^ [kg]

The final optimised parameter values, which yielded the closest agreement with the experimental data, are summarised as follows:

*k* = 1.6304 × 10^3^ [N/m]

*b* = 316.2262 × 10^3^ [kg/s]

Highlighting that such experimentally validated values are rarely reported in the literature, where most studies rely on theoretical assumptions or general manufacturer specifications.

## 5. Conclusions

The conducted research, both experimental and numerical, provided valuable information on the properties of MEMS sensors and confirmed the effectiveness of combining modern analytical methods and modelling in their evaluation. The use of SEM electron microscopy and EDS allowed for a detailed analysis of the sensor’s microstructure and chemical composition. The results obtained enabled a thorough understanding of the impact of these factors on the mechanical properties of the sensor, which was crucial for further research. The development of a numerical model of the MEMS sensor in the MATLAB Simulink environment allowed for detailed simulations that reproduced the actual behaviour of the sensor in response to various mechanical stimuli. The use of the numerical model enabled the analysis of key parameters such as stiffness, damping and response to forces, which allowed for the optimisation of the sensor design. The designed workstation enabled accurate monitoring of the sensor’s response to mechanical forces under various conditions. The measurement results were compared with the results obtained from numerical simulations, which allowed for calibration and further refinement of the model. Numerical tests carried out on a system with a higher sampling frequency (65 kHz) showed high consistency with actual measurements. The differences between the results were minimal, mainly due to numerical rounding, which did not affect the overall quality of the model. A comparison of different data filtering methods showed that the proposed filtering system is characterised by higher precision and better reproduction of reference results, which highlights its advantages in the analysis of results obtained from MEMS sensors. The combination of experimental results with numerical modelling enabled the sensor to be precisely adjusted to actual operating conditions. The tools used allowed conclusions to be drawn about the strength of materials, their mechanical properties, and the prediction of potential damage during use. A comprehensive approach, combining experimental analyses with advanced numerical simulations, allowed for the effective optimisation of MEMS sensor recorders, improving quality and reliability and simplifying the research preparation process. In light of the above considerations, the subsequent research will concentrate on the development of an electromechanical model that incorporates the capacitive behaviour of the sensor, including the variable geometry of the capacitors.

## Data Availability

The original contributions presented in this study are included in the article. Further inquiries can be directed to the corresponding author.

## Abbreviations

The following abbreviations are used in this manuscript:

EDS	Energy Dispersive Spectroscopy
SEM	Scanning Electron Microscope
MEMS	Microelectromechanical Systems
